# Characterizing the Neurodevelopmental Pesticide Exposome in a Children’s Agricultural Cohort

**DOI:** 10.3390/ijerph17051479

**Published:** 2020-02-25

**Authors:** Breana Bennett, Tomomi Workman, Marissa N. Smith, William C. Griffith, Beti Thompson, Elaine M. Faustman

**Affiliations:** 1 Department of Environmental and Occupational Health Sciences, School of Public Health, University of Washington, Seattle, Washington, DC 98105, USA; benneb3@uw.edu (B.B.); workmt@uw.edu (T.W.); marissa.nicole.smith@gmail.com (M.N.S.); griffith@uw.edu (W.C.G.); 2 Institute for Risk Analysis and Risk Communication, University of Washington, Seattle, Washington, DC 98105, USA; 3 Public Health Sciences, Fred Hutchinson Cancer Research Center, Seattle, Washington, DC 98105, USA; bthompso@fredhutch.org

**Keywords:** dust analysis, environmental exposure analysis, farmers, longitudinal studies, pesticides analysis, neurodevelopment, exposome, occupational exposure analysis

## Abstract

The exposome provides a conceptual model for identifying and characterizing lifetime environmental exposures and resultant health effects. In this study, we applied key exposome concepts to look specifically at the neurodevelopmental pesticide exposome, which focuses on exposures to pesticides that have the potential to cause an adverse neurodevelopmental impact. Using household dust samples from a children’s agricultural cohort located in the Yakima Valley of Washington state, we identified 87 individual pesticides using liquid chromatography-tandem mass spectrometry. A total of 47 of these have evidence of neurotoxicity included in the Environmental Protection Agency (EPA) (re)registration materials. We used a mixed effects model to model trends in pesticide exposure. Over the two study years (2005 and 2011), we demonstrate a significant decrease in the neurodevelopmental pesticide exposome across the cohort, but particularly among farmworker households. Additional analysis with a non-parametric binomial analysis that weighted the levels of potentially neurotoxic pesticides detected in household dust by their reference doses revealed that the decrease in potentially neurotoxic pesticides was largely a result of decreases in some of the most potent neurotoxicants. Overall, this study provides evidence that the neurodevelopmental pesticide exposome framework is a useful tool in assessing the effectiveness of specific interventions in reducing exposure as well as setting priorities for future targeted actions.

## 1. Introduction

### 1.1. The Exposome

The concept of the exposome [1] provides an ambitious model for identifying and characterizing lifetime environmental exposures and resultant health effects. Environmental exposures, including diet, behavior, and exogenous and endogenous (e.g., hormones) factors, account for the majority of disease risk [2]; thus, the exposome is an important complement to the genome in terms of understanding human health. However, the wide variety of environmental factors, biological endpoints, and gene–environment interactions make the task of defining the human exposome difficult [1,2,3,4,5,6,7,8,9]. Wild [1] suggested that there were three broad exposure categories—internal (e.g., hormones, microflora), specific external (e.g., infectious disease, toxicants), and general external (e.g., social, psychological)—and that measuring in any one area can reflect certain aspects of the overall exposome [6]. Several methods for evaluating the exposome have been proposed, including what Rappaport and Smith [4] termed “bottom-up” environmental monitoring and “top-down” biomonitoring, the latter of which has received much attention for its potential for revealing both internal and external components of the exposome [4]. However, biomonitoring alone can be difficult to connect to specific exposures, which can make risk assessment and intervention, including regulatory decision making, difficult [6]. Thus, for the purposes of protecting human health, it can be advantageous to narrow the focus of the exposome to a particular class of exposures, such as pesticides, as a way to apply broader exposome concepts in a risk assessment or regulatory framework. Additionally, focusing the exposome on specific life stages (e.g., specific periods of development) can both narrow down the compounds of interest and the window of exposure monitoring [10,11], especially for episodic exposures, such as agricultural pesticides that are typically used on a seasonal basis. Focusing specifically on classes of exposures or specific life stages allows the broader exposome concepts to have more utility in regulation or decision making. 

### 1.2. Aggregate Exposure Pathways (AEPs) and the Exposome

Teeguarden et al. [12] have proposed using an aggregate exposure pathway (AEP) as an organizational framework connecting exposure science to environmental health. The AEP builds on the concept of the adverse outcome pathway (AOP), which is a framework that links molecular perturbations to an adverse outcome of regulatory relevance. Using the same basic concept, the AEP links the introduction of a stressor into the environment to its ultimate concentration at a biologically relevant site of action. In this framework, Teeguarden et al. define exposure as the actual amount of stressor reaching the environmental area of interest (e.g., buildings, soil, human tissues) without determining when that exposure becomes a dose [12]. This AEP framework provides a possible avenue for narrowing the focus of the broader exposome concept while allowing the exposome to reflect important complex exposure profiles. By looking at a class of stressors, such as pesticides, and how they accumulate at a specific site of action, the exposome can be more directly applied in a framework that is better suited for regulatory decision making and other related public health interventions. 

### 1.3. The Neurodevelopmental Exposome

Neurodevelopment is a critical endpoint to examine in children’s health [13]. Although the majority of neurogenesis and neuron migration occurs during the prenatal period, there are still key processes in brain development that occur postnatally [14]. As a result, there is an extended “window of susceptibility” for children’s neurodevelopment, and exposure to environmental neurotoxicants throughout early adolescence can have significant impacts on a child’s learning, behavior, and attention [13,14,15,16]. There are a number of well-characterized environmental toxicants that are known to have an adverse impact on children’s neurodevelopment, including lead, methylmercury, tetrachloroethylene, and the organophosphate (OP) pesticide chlorpyrifos [15]. As children can be exposed to a number of environmental neurotoxicants at any given time, it is important to consider the neurodevelopmental exposome when assessing the potential adverse health effects of child exposures, especially to pesticides. 

Many pesticides share similar modes of action, which can exacerbate the impacts on human health when co-exposures occur. For example, OP and N-methyl carbamate pesticides both act by inhibiting acetylcholinesterase, thus co-exposures can lead to an even greater accumulation of acetylcholine and subsequent neurotoxic effects. The neurodevelopmental exposome follows on this concept by considering multiple pesticide classes that may act by different modes of action but share similar target systems such as the neurological system. In 2011, a trio of child health studies observed significant neurodevelopmental delays associated with prenatal and early childhood exposure to OPs [17,18,19]. Other pesticide groups, such as the neonicotinoids have also been associated with impaired neurodevelopment in animal models [20]. Both OPs and neonicotinoids are commonly used in agriculture, and so, exploring the neurodevelopmental pesticide exposome is particularly important when considering the health of children living in complex and dynamic agricultural communities.

### 1.4. Applying a Neurodevelopmental Exposome Framework to Evaluate the Potential Health Impacts of Pesticide Regulation

In this example, we will use our previous work in the Yakima region of Washington state with a longitudinal children’s cohort focused on families in this agricultural community in which we have evaluated temporal patterns of pesticide use and exposure [1]. In Washington state, apples are the number one commodity produced in the USD 49 billion food and agriculture industry [21,22]. In order to maximize production, apple growers rely on pesticides to control insect, plant, and fungal pests [23]. Historically, azinphos-methyl (AZM) was among the most commonly used OPs in agriculture, particularly in apple production, but due to concerns regarding worker health and ecological impacts, a phase-out of all AZM uses was issued by the Environmental Protection Agency (EPA) in 2006 [24,25]. This phase-out concluded with AZM sales being prohibited in 2012 and the use of any existing stocks prohibited in 2013 [25]. Pome fruits (apples and pears) were among the last allowable uses for AZM, in part because of the heavy reliance on AZM for control of codling moth, one of the major apple pests [26].

One of the major reasons for the incremental phase-out of AZM was to minimize the negative economic impacts of eliminating a major method of pest control by allowing sufficient time for growers to transition to alternative chemicals and methods of insect control [24,25,26]. Throughout the duration of the AZM phase-out, Washington State University (WSU) Extension issued reports on suitable alternatives to AZM to help control codling moth. The alternatives to AZM suggested by WSU Extension include other OPs such as phosmet and diazinon, neonicotinoids such as acetamiprid and thiacloprid, N-methyl carbamate carbaryl, and insect growth regulators methoxyfenozide and pyriproxyfen [26,27]. Given that the OPs, N-methyl carbamates, and neonicotinoids all target the nervous system, using an exposome framework could provide a unique avenue to evaluate the impact of the AZM phase-out on pesticide exposure and potential adverse health outcomes, particularly in regards to children living in these agricultural communities. Under the Food Quality Protection Act (FQPA), the EPA must consider the cumulative health effects of exposure to pesticides with a common mechanism of toxicity, defined as “two or more pesticide chemicals or other substances that cause a common toxic effect to human health by the same, or essentially the same, sequence of major biochemical events”. However, these cumulative assessments are not applied across pesticides with common health endpoints.

### 1.5. Using a Neurodevelopmental Exposome Framework to Characterize Pesticide Exposure in a Children’s Agricultural Cohort

In the present study, we have combined key concepts from the AEP and exposome frameworks to characterize the neurodevelopmental pesticide exposome in a longitudinal children’s agricultural cohort located in the Lower Yakima Valley of Washington state. By using household dust as a representative exposure medium, we have determined the levels of prevalent pesticides used in pome fruit (apples and pears) production in the Yakima Valley for two agricultural seasons six years apart (2005 and 2011). In particular, we focused on a timepoint prior to the AZM phase-out (2005) and near the end of the phase-out (2011) to assess how substitutions away from AZM affected the overall neurodevelopmental pesticide exposome. This approach of using pesticide levels in household dust as an indicator of child pesticide exposure is supported by dust correlations of urinary biomarkers and dust pesticide levels in previous work published on this cohort [28,29,30]. Furthermore, there is strong evidence that changes in the use of agricultural pesticides is reflected in the household dust of farmworker households [31].

## 2. Materials and Methods 

### 2.1. Cohort Description and Sample Collection 

The University of Washington Center for Child Environmental Health Risks Research (CHC) cohort is an agricultural children’s cohort located in the Lower Yakima Valley. Since 1998, the CHC has worked with 800 families (beginning with the “Para Niños Saludables” cohort [32]). The present study examines a nested cohort of approximately 200 households split approximately evenly between farmworker (FW) and non-farmworker (NFW) families, each with a referent child aged 2–6 years. Of these 200 households, 75 had dust samples collected between April and July of 2005. In 2011 dust samples were collected between June and August from a smaller subset of the households: 60 farmworker and 40 non-farmworker households. Dust samples were collected by vacuuming 0.5 m × 0.5 m square areas of floor surface as previously described by Smith et al., 2016 [33]. 

### 2.2. Dust Analysis

Dust analysis methods were applied in this study consistent with our previously published methods [32,33,34,35,36,37,38,39]. Dust samples were prepared by sieving the dust through 150 µm metal sieves for 10 min and partitioning the remaining dust into 1 g aliquots. Samples were sonicated for 1 min at 20 kHz using a horn-type cell disrupter after the addition of acetone (10 mL) and then centrifuged for 5 min at 3000 rpm. The supernatant (8.0 mL) was transferred to a 50 mL turbovap tube and evaporated to less than 1 mL at 45 °C. After this initial evaporation, samples were vortexed, washed with 3–4 mL of cyclohexane, and evaporated to less than 1 mL at 45 °C. The volume of each sample was standardized by increasing the volume to 1 mL by adding cylcohexane, and then, 2.5 mL of 20% dichloromethane in cyclohexane was added. Samples were centrifuged at 3000 rpm for 10 min. Cleanup of the dust extracts was completed using gel permeation chromatography (GPC) with high performance liquid chromatography (HPLC) and Waters Sample Manager programmed to inject 3.5 mL of sample with a run time of 55 min. Collection time was 21–55 min. Fractions were evaporated to a volume less than 1 mL by using the sensor endpoint with 14 psi nitrogen at 60 °C. The sides of the Turbovap flask were rinsed with 4–5 mL Trimethyl phosphate (TMP), and the sample was evaporated again to the sensor endpoint to leave a final volume of slightly less than 0.5 mL. Samples were resuspended to the 0.5 mL mark using TMP. All samples were filtered with a 0.45 μm syringe filter before being transferred into a 2 mL GC vial. Vials were stored at −15 °C until analysis.

HPLC-tandem mass spectrometry (HPLC-MS/MS) analysis was accomplished by using stable isotope-dilution quantification. Agilent 6410 HPLC-MS/MS was operated in positive electrospray ionization (ESI+) and multiple reaction mode (MRM) with nitrogen collision gas (Gas temp: 350 °C; gas flow: 9 L/minute; nebulizer: 40 psi; capillary voltage: 4000 V). A subset of compounds (2,4-D; 2,4-DP; MCPA; MCPP; 2-phenyl phenate; triclosan) was quantified by negative electrospray ionization (ESI−). These methods were previously described in [32,33].

The HPLC system was equipped with a Gemini (Phenomenex) C18 reverse-phase column (3 micron, 150 × 2.0 mm), with a Gemini 4 × 2.0 mm guard column. All solvents (HPLC-grade) and deionized water (Barnstead Nanopure II, 18 MΩ) used were monitored for background and included procedural blanks. Details on the instrument parameters can be found in Armstrong et al., 2014. A total of 87 pesticides out of 145 initial candidates were successfully analyzed (as described by Bennett et al. [31]). 

### 2.3. Assessment of Potential Pesticide Neurotoxicity

All 87 pesticides that were detected in the household dust samples were included in the neurotoxicity assessment. For each pesticide, the neurotoxicity potential was initially scored by a simple “yes/no” system based on the data available in U.S. EPA Registration Eligibility Decisions (REDs). All pesticide REDs were searched for using the EPA Pesticide Chemical Search Database (https://iaspub.epa.gov/apex/pesticides/f?p=chemicalsearch:1) [40]. For pesticides without a RED available at the time of analysis (e.g., boscalid), the most recent human health risk assessment or other relevant health assessment material available in the pesticide registration (or reregistration review) docket was used instead. Pesticides were scored as “potentially neurotoxic” if the RED (or other available material) provided a specific dose (e.g., LOAEL, NOAEL, RfD, etc.) associated with a neurotoxic endpoint. Neurotoxic endpoint assessment followed the EPA guidelines for neurotoxicity risk assessment [41]. Briefly, five broad endpoints were used to identify neurotoxic effects: structural (e.g., morphological changes in the brain), neurophysiological (e.g., alterations in nerve conduction), neurochemical (e.g., alterations in synthesis/breakdown of neurotransmitters), behavioral (e.g., changes in motor activity), and developmental (e.g., delayed behavior appearance) endpoints [41]. Pesticides were scored as “non-neurotoxic” if the RED or other available (re)registration documents did not conduct a neurotoxicity evaluation or include any neurotoxic endpoints. Dose information was used from the “Summary of Toxicology Endpoint Selection” for non-occupational exposures available in the RED or human health risk assessment. For weighted analyses (i.e., accounting for relative neurotoxic potency), the levels of each potentially neurotoxic pesticide detected in the household dust were divided by the reference dose (RfD) provided in the (re)registration materials. 

### 2.4. Statistical Analysis

#### 2.4.1. Heat Map Generation

**Figure 1:**
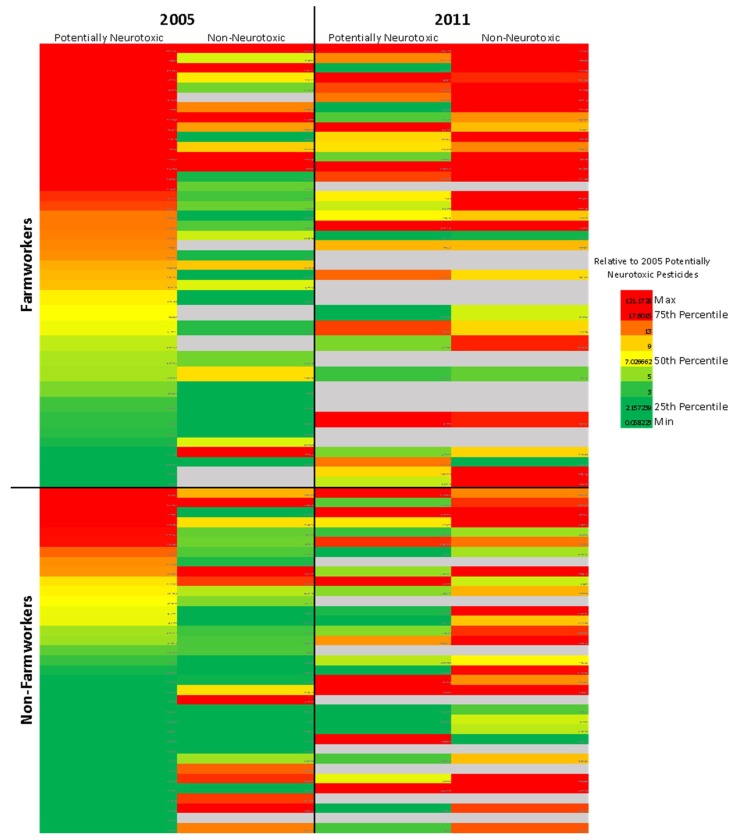
Neurodevelopmental pesticide exposome heat map. Using the pesticide categorizations provided in Table 1 and Table 2, the concentrations of potentially neurotoxic and non-neurotoxic pesticides (in µmol/g dust) were summed for each household (row). Figure 1 shows how the levels of potentially neurotoxic and non-neurotoxic pesticides changed over the two sampling periods (2005 and 2011) and different between farmworker and non-farmworker households. The red coloring indicates pesticide levels greater than the 75th percentile for potentially neurotoxic pesticides in 2005 (17.6 µmol/g), yellow indicates the 50th percentile (7.03 µmol/g), and green indicates the 25th percentile (2.16 µmol/g).

A neurodevelopmental exposome heat map was generated using pesticide levels detected in household dust (see Figure 1). This method of heat map generation was adapted from the method described by Smith et al. [33]. The columns represent the grouped potentially neurotoxic or non-neurotoxic pesticides for each of the study years (2005 and 2011), and each row represents an individual household. In order to explain the complexities of the pesticide exposures over these years, we compared all years to the amount of pesticide exposure occurring in the first sampling period by coding the amount as detailed below. A tri-color (red, yellow, green) scoring system was used to relate each group of pesticides to the concentration of neurotoxic pesticides detected in 2005. The red coloring represents pesticide levels greater than the 75th percentile for 2005 neurotoxic pesticides (17.60 µmol/g dust), yellow represents the 50th percentile (7.03 µmol/g dust), and green represents the 25th percentile (2.16 µmol/g dust). This same coloring system was applied to both groups of pesticides for both study years to allow for comparisons across time. 

#### 2.4.2. Farmworker vs. Non-farmworker Comparisons

A binomial comparison was used to assess the difference between the levels of neurotoxic and non-neurotoxic pesticides detected in the household dust between FW and NFW households, as previously described by Bennett et al. [31]. Using the heat map coloring (described above), the proportion of “red” households (i.e., greater than 17.60 µmol/g dust) was determined for each pesticide grouping. This generated two categories (i.e., high vs. low concentration). A nonparametric proportions test was then used to compare the redness of FW versus NFW houses. This allowed us to determine whether FWs or NFWs have a higher proportion of household dust samples with the highest levels of potentially neurotoxic pesticides and whether those differences were consistent between 2005 and 2011. This analysis was conducted using both the unweighted and weighted levels of potentially neurotoxic pesticides.

#### 2.4.3. Comparisons across Time

A mixed effects model was used to examine the trends in potentially neurotoxic and non-neurotoxic pesticides between the two study years (2005 and 2011). The mixed effects model used for this analysis was the following:

Log(Y) = a + b(t) + c(occ) + d(t * occ) + R(σ_b_) + E(σ_w_)

where Y is the sum of all pesticide levels within a given neurotoxicity grouping (i.e., potentially neurotoxic vs. non-neurotoxic; in µmol); a is the 2005 FW average pesticide level, b is the coefficient for the time adjustment (2011 relative to 2005); t is time; c is the coefficient for the occupation adjustment (NFW relative to FW); occ is occupation; d is the coefficient for the interaction adjustment for time and occupation; R is the random effect to account for between-household variability (measured by σb); and E is the within-household variability (measured by σw). Equation (1) allows us to determine whether time had an effect on potentially neurotoxic pesticide levels detected in the household dust and whether those changes in time were affected by occupation. An additional model looked at the effect of time within each occupation individually, which allowed us to see whether potentially neurotoxic pesticide levels changed with time for each occupation independently. These analyses were conducted using the unweighted potentially neurotoxic pesticide levels, the non-neurotoxic pesticide levels, and the weighted potentially neurotoxic pesticide levels. 

An additional non-parametric binomial analysis was used to assess whether the change in potentially neurotoxic and non-neurotoxic pesticides between 2005 and 2011 was significantly different between FWs and NFWs. For each household, we determined whether the levels of potentially neurotoxic/non-neurotoxic pesticides increased, decreased, or stayed the same between 2005 and 2011. Then, the proportion of households whose levels decreased over time was determined for each occupation (FW vs. NFW), and a nonparametric proportions test was used to determine whether the proportion of households with decreased pesticide levels was significantly different between FWs and NFWs. This analysis was conducted using both the unweighted and weighted levels for the potentially neurotoxic pesticides. 

## 3. Results

### 3.1. Categorization of Potentially Neurotoxic Pesticides

Table 1 details which pesticides have neurotoxic potential, as determined by EPA REDs, human health risk assessments, or other available pesticide (re)registration information. Of the 87 initial pesticides, 47 had some evidence of neurotoxicity in animal models used in the EPA (re)registration decision materials. The remaining 39 pesticides either had no neurotoxicity assessments conducted in the (re)registration process or had no neurotoxic endpoint in the toxicology endpoint summary table. For pesticides that did have a neurotoxic endpoint provided in the (re)registration materials, the associated dose information is provided in Table 1. When available, the acute reference dose (RfD) or population adjusted dose (PAD) was selected. Otherwise, the most sensitive acute dose (e.g., NOAEL used over LOAEL) was selected, and the RfD was calculated using standard uncertainty factors of 100× for NOAELs and 1000× for LOAELs. 

### 3.2. Neurodevelopmental Exposome Heat Map

Figure 1 shows the concentration of potentially neurotoxic and non-neurotoxic pesticides for both study years (2005, left, and 2011, right) among farmworkers (top) and non-farmworkers (bottom). The red coloring indicates pesticide levels that are greater than the 75th percentile of potentially neurotoxic pesticides in 2005 (17.60 µmol/g dust).

### 3.3. Trends in Potentially Neurotoxic Pesticides

#### 3.3.1. Unweighted Analysis

**Figure 2:**
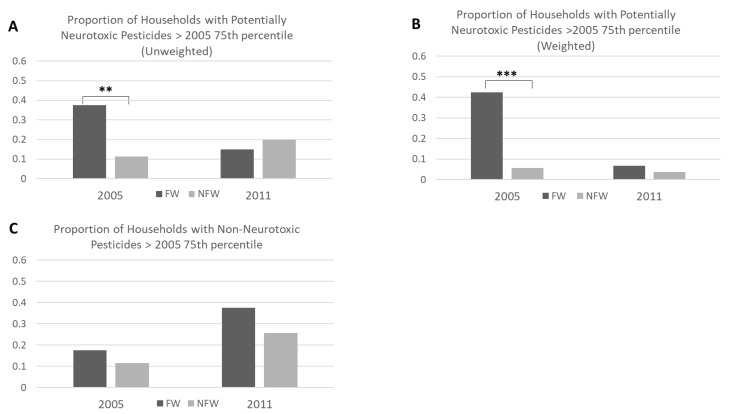
Proportion of households with high pesticide concentrations, 2005–2011. Figure 2 shows the proportion of farmworker (FW) and non-farmworker (NFW) households that had high (>17.6 µmol/g) potentially neurotoxic pesticide levels (unweighted analysis in panel A, weighted analysis in panel B) and non-neurotoxic pesticide levels (panel C) in 2005 and 2011. Black bars represent FW households, and grey bars represent NFW households. ** *p* < 0.01; *** *p* < 0.001.

Figure 2A shows that in 2005, 38% of FW households had potentially neurotoxic pesticide levels greater than the 75th percentile (17.60 µmol/g dust, colored red in Figure 1), which is significantly greater than the 11% of “red” NFW households (Table 2, difference = 0.27 (95% CI: 0.08–0.44), *p* = 0.01). By 2011, the proportion of red households was not significantly different between FWs and NFWs (Table 2, *p* = 0.57). Across the entire cohort, the levels of potentially neurotoxic pesticides detected in the household dust were not significantly different between 2005 and 2011 (Table 2, *p* = 0.57). However, when occupation was added into the model (with interaction), the difference between 2005 and 2011 was statistically significant (Table 2, *p* > 0.001). This suggests that the levels of potentially neurotoxic pesticides for FWs and NFWs change differently over the study period. Indeed, there was a higher proportion of NFW households that had high levels of potentially neurotoxic pesticides in 2011 than in 2005, although this difference is not statistically significant (*p* = 0.28). 

#### 3.3.2. Weighted Analysis

Figure 2B shows that in the weighted neurodevelopmental pesticide exposome analysis, 43% of FWs had households with potentially neurotoxic pesticide levels greater than the 75th percentile (weighted), which is significantly greater than the 6% of NFW households (Table 3, difference = 0.37 (95% CI: 0.20–0.54), *p* < 0.001). In 2011, the proportion of households above the 2005 75th percentile was not significantly different between FWs and NFWs (Table 3, *p* = 0.62). Across the entire cohort, the levels of potentially neurotoxic pesticides decreased between 2005 and 2011 (Table 3, *p* = 0.004), but this decrease was only significant among FWs (*p* = 0.001) and not among NFWs (*p* = 0.38). Additionally, the trend of increasing potentially neurotoxic pesticide levels among NFW households that was present in the unweighted analysis does not appear in the unweighted analysis. 

### 3.4. Trends in Non-Neurotoxic Pesticides

Figure 2C shows that for both 2005 and 2011, the proportion of FW households that had non-neurotoxic pesticide levels greater than the 75th percentile of potentially neurotoxic pesticides (17.60 µmol/g dust, colored red in Figure 1) was not significantly different than the proportion of red NFW households (Table 2, *p* = 0.46 for 2005, *p* = 0.28 for 2011). Across the entire cohort, the levels of non-neurotoxic pesticides in the household dust were significantly higher in 2011 than in 2005 (*p* < 0.001). This same trend was observed among FWs and NFWs individually as well (Table 2, *p* < 0.001). Additionally, there was no significant difference between the proportions of FW vs. NFW households with decreased non-neurotoxic pesticide levels between 2005 and 2011 (*p* = 0.36).

## 4. Discussion

### 4.1. The Neurodevelopmental Exposome of Farmworkers and Non-farmworkers became More Similar over Time

In 2005, FW households tended to have significantly higher levels of potentially neurotoxic pesticides than NFW households in both the weighted and unweighted analyses. However, by 2011, there was no significant difference between the two occupational groups in either analysis. These differences between FW and NFW households are likely largely driven by the OPs, all of which have a neurotoxic endpoint (AChE inhibition) and decreased significantly between 2005 and 2011 [33]. Most OPs are restricted to agricultural uses, which is consistent with the observation that the decline in potentially neurotoxic pesticides mirrors the decline in OPs for FWs, but not for NFWs. Furthermore, there was an increase in the levels of non-neurotoxic pesticides detected in the household dust for both occupational groups. This suggests that there may be a shift in pesticide use away from potentially neurotoxic pesticides towards non- or less-neurotoxic alternatives. Indeed, the regulatory efforts of the EPA reflect a movement away from neurotoxic pesticides in apple production [24,42,43,44].

It should be noted, however, that in the present study neurotoxicity was assessed by the health endpoints highlighted in the EPA REDs. The “non-neurotoxic” pesticides may still have an effect on the nervous system that has either not been well characterized or was not sensitive enough to be incorporated into the EPA (re)registration decision. Additionally, this analysis did not include pesticides that may affect the neuroendocrine system in the potentially neurotoxic classification. For example, according to the EPA Human Health Assessment Scoping Document, the anilide fungicide boscalid does not directly affect the nervous system but has been reported to affect the thyroid [44]. For this analysis, these pesticides were categorized as “non-neurotoxic”, because it is difficult to assess whether alterations to the endocrine system will ultimately affect the nervous system. Instead, this analysis focused on pesticides that have overt neurotoxicity to allow for clearer endpoint definition. Future studies may include potential neuroendocrine effects in the neurodevelopmental exposome, but that assessment is beyond the scope of this analysis. 

### 4.2. Weighted Analysis Reveals a Greater Decline in the Levels of More Potent Potentially Neurotoxic Pesticides with Time

Although the neurodevelopmental exposome of FWs and NFWs became more similar over time, a mixed effects analysis suggests that these exposomes are changing differently with time. In the unweighted analysis, the FW households tended towards having decreased levels of potentially neurotoxic pesticides (non-significant), whereas the NFW households tended towards increasing levels (non-significant). The trend of decreasing levels of potentially neurotoxic pesticides among FWs is consistent with decreasing OP use in apple production [33], but the increasing trend among NFWs was unexpected. When the potentially neurotoxic pesticides were weighted by their relative potencies, the trend of decreasing potentially neurotoxic pesticides does become significant for FWs, and, while still non-significant, the trend towards increasing potentially neurotoxic pesticide levels disappears among NFWs. These differences between the weighted and unweighted analysis suggest that the pesticides that were decreasing the most between 2005 and 2011 were those that are more potent (i.e., have lower RfDs). This is consistent with the significant decrease observed in the OPs across the entire cohort over this study period [33]. The OPs were among some of the more potent neurotoxicants (see Table 1) and were present in very high levels in 2005 but decreased significantly by 2011 [33]. The phase-out of AZM that was occurring during this study period was likely contributing to these changes. By using both the weighted and unweighted analyses, we can demonstrate that not only were potentially neurotoxic pesticide levels decreasing over this study period, but the levels of some of the most potent potentially neurotoxic pesticides were also decreasing. This provides evidence that the changes in pesticide use, whether driven by regulatory or other pressures, were largely focused on the more potent neurotoxicants. 

### 4.3. Limitations

As previously discussed, this analysis was focused solely on potentially neurotoxic health effects as defined by the EPA guidelines for neurotoxicity risk assessment [41], which does not include neuroendocrine effects. In recent years, the links between the endocrine system and neurodevelopmental process, in particular, have become more well defined [45]. Thus, this analysis may not provide the full scope of potential neurodevelopmental toxicants present in the pesticide exposome for this cohort. Broadening decision criteria to include endocrine disruption would be excellent for future work but as a potentially neurotoxic endpoint would greatly complicate the analysis, as determining which endocrine pathways ought to be included and assessing the overall potency of endocrine disruptors on neurodevelopment is complex. Furthermore, any potential interactions (i.e., synergic or antagonistic effects) between pesticides were not evaluated in the present study, as in this study, we chose to evaluate additivity, consistent with current regulations in the U.S. Another limitation to this study stems from using REDs and other U.S. EPA (re)registration materials as data sources. This resulted in two potential limitations. For this first evaluation, we chose to evaluate the pesticide exposures with reference to their acute toxicological impacts. This is supported by the fact that many of these pesticides have short half-lives, many less than 36 hours. Hence, repeated exposures are of interest, but many differ toxicologically from chronic exposure endpoints. Additional assessment of these pesticides, which have chronic toxicity information, would be interesting for future studies. Another limitation for the REDs is that the process of updating (re)registration materials is slow and labor intensive. Thus, many of the materials used to identify neurotoxic endpoints in this study may not fully reflect the current evolving research on these pesticides. For example, several neonicotinoid insecticides are currently undergoing the reregistration process, but those updates have yet to be released to the public [46]. On the other hand, by using EPA (re)registration materials, there is the benefit of consistency in the development of RfDs and endpoint selection that provides us with more confidence that our assessment of potential neurotoxicity is unbiased. The constructs that we have established in this paper will allow for these future assessments.

## 5. Conclusions

This study looks at multiple pesticide exposures in an exposome framework. By looking at a wide variety of pesticides with a common target organ system, we can gain a better understanding of how changes in exposure may affect the risk of specific health outcomes. Although there were no significant changes in the neurodevelopmental pesticide exposome between 2005 and 2011 in the unweighted analysis, weighting each potentially neurotoxic pesticide by its RfD reveals a significant decrease in the levels of potentially neurotoxic pesticides detected in the household dust across the entire cohort. This trend was largely driven by FW households and was largely due to the significant decrease in exposure to OPs over this study period. These findings are consistent with regulatory efforts by the U.S. EPA to reduce exposures to OPs during the timeframe of this study. Furthermore, this study provides evidence that the more potent potentially neurotoxic pesticides were reduced more than some of the less potent potentially neurotoxic pesticides over the study period, suggesting that the substitutes for AZM in apple production have less neurotoxic potential than AZM. Given that the AZM phase-out was largely prompted by worker health concerns, the decrease in potentially neurotoxic pesticides over this study period provides supporting evidence that the phase-out was effective in that regard. Thus, this study provides evidence that using a neurodevelopmental pesticide exposome framework could be a useful tool in assessing the effectiveness of specific interventions in reducing exposure to neurotoxicants. Our intervention research has shown that for organophosphate pesticides we can successfully reduce the pesticides in household dust, which provides promise for extending these interventions to address the overall pesticide exposome [47]. This novel application of exposome concepts in a regulatory framework provides a new avenue to further explore the utility of the broader exposome framework for evaluating changes in exposure linked with risks for neurotoxicity. 

## Figures and Tables

**Table 1:** Classification of pesticides detected in household dust. Pesticides were identified as potentially neurotoxic based on available health endpoint data in U.S. Environmental Protection Agency (EPA) Registration Eligibility Decisions (REDs), human health risk assessments, or other health assessment data available in (re)registration dockets. Table 1 identifies which pesticides were identified as potentially neurotoxic and provides a reference dose (RfD) for each.

Compound	Identified as Neurotoxic	Reference Dose (mg/kg/Day)	Compound	Identified as Neurotoxic
2_4D	Y	0.067	2_4DB	N
Acetamiprid	Y	0.1	2_4DP	N
Aldicarb	Y	0.001	Boscalid	N
Azinphosmethyl	Y	0.0033	Carfentrazone-ethyl	N
Azoxystrobin	Y	0.67	Clofentezine	N
Bifenazate	Y	0.134 *	Diuron	N
Carbaryl	Y	0.01	Dodine	N
Carbofuran	Y	0.00006	Etoxazole	N
Chlorpyrifos	Y	0.005	Fenarimol	N
Clothianidin	Y	0.025	Fenhexamid	N
Coumaphos	Y	0.007	Fenoxycarb	N
Cyphenothrin	Y	0.23 *	Fenpyroximate	N
Deltamethrin	Y	0.01	Flumioxazin	N
Diazinon	Y	0.0025	GibberellicAcid	N
Dicamba	Y	1	Hexythiazox	N
Dichlorvos	Y	0.008	Imazamox	N
Dimethoate	Y	0.013	Imazapic	N
Ethoprop	Y	0.00025	Imazapyr	N
Etofenprox	Y	0.57 *	Imazethapyr	N
Imidacloprid	Y	0.14	Linuron	N
Imiprothrin	Y	0.33 **	Mefenoxam	N
Malathion	Y	0.14	Metribuzin	N
MCPA	Y	0.04	Myclobutanil	N
MCPP	Y	1.75	Na o Phenylphenate	N
Methamidophos	Y	0.003	Norflurazon	N
Methidathion	Y	0.002	Novaluron	N
Methomyl ^	Y	0.05 *	Pendimethalin	N
Methyl Parathion	Y	0.0011	Piperonyl Butoxide	N
Naled	Y	0.01	Propargite	N
Oxamyl	Y	0.001	Pyrimethanil	N
Permethrin	Y	0.25	Pyriproxyfen	N
Phorate	Y	0.0025	Quinclorac	N
Phosmet	Y	0.045	Quinoxyfen	N
Pirimicarb	Y	0.04 *	S-Metolachlor	N
Propiconazole	Y	0.3	Spinosyn A/D (spinosad)	N
Propoxur	Y	0.005	Triclopyr	N
Pyridaben	Y	0.44	Triclosan	N
S-Bioallethrin ^^	Y	0.0013 ***	Trifloxystrobin	N
Sumithrin	Y	0.03	Tetramethrin	N
Tebuconazole	Y	0.016 #		
Terbufos	Y	0.0003		
Tetrachlorvinphos	Y	0.067		
Thiacloprid	Y	0.01		
Thiamethoxam	Y	0.35		
Thiophanate methyl	Y	0.4		
Triadimefon	Y	0.034		
Triflumizole	Y	0.25		

Acute dietary exposure was the route of exposure unless otherwise indicated. For pesticides with a Population Adjusted Dose (PAD), the additional Food Quality Protection Act (FQPA) safety factor was multiplied back out for consistency with reported reference doses. In cases where different doses were given for males and females, the more sensitive population was used. * RfDs marked by asterisks were calculated from NOAELs reported in the available docket information. Standard UFs of 100 were applied in each case. ** RfD was calculated from the reported NOAEL of 100 mg/kg/day and divided by the UFs of 300, as reported in the RED. *** RfD was calculated from the reported NOAEL of 1.3 mg/kg/day and divided by the UFs of 1000, as reported in the RED. # RfD was calculated from the reported LOAEL of 16.3 mg/kg/day and divided by standard UFs of 1000. ^ The only route of entry with a neurotoxic endpoint was dermal. ^^ The only route of entry with a neurotoxic endpoint was inhalation.

**Table 2:** Statistical summary table for unweighted neurodevelopmental exposome analysis. Proportion tests: for both neurotoxicity groupings, the proportion of “red” FW households in Figure 1 (levels greater than 17.6 µmol/g) was compared with the proportion of red NFW households for both study years (2005 and 2011). Table 2 provides the proportion of red FW households, the proportion of red NFW households, the difference between the two, and the associated p-value. Additionally, the proportion of FW vs. NFW households with decreased pesticide levels was compared for both study years. Mixed effects tests: Table 2 also provides the mixed effects models used to examine cohort-wide trends in potentially neurotoxic and non-neurotoxic pesticides over time, which models were compared in the analysis, and whether those models were significantly different from one another. * *p* < 0.05; *** *p* < 0.001.

	Potentially Neurotoxic Pesticides				Non-Neurotoxic Pesticides			
Proportions Tests	FW	NFW	Difference (95% CI)	*p*	FW	NFW	Difference (95% CI)	*p*
Proportion Red 2005	0.38	0.11	0.27 (0.08–0.44)	0.01 *	0.18	0.11	0.07 (−0.10–0.22)	0.46
Proportion Red 2011	0.15	0.20	−0.5 (−0.22–0.12)	0.57	0.38	0.26	0.12 (−0.09–0.33)	0.28
Proportion Decreased (2005–2011)	0.70	0.52	0.18 (−0.07–0.43)	0.16	0.25	0.15	0.1 (−0.12–0.32)	0.36
	**Potentially Neurotoxic Pesticides**				**Non-Neurotoxic Pesticides**			
**Mixed Effects Tests**	**Models**			***p***	**Models**			***p***
Null vs. Time Fixed	log(pest) ~ 1 + (1|house) log(pest) ~ tm + (1|house)			0.75	log(pest) ~ 1 + (1|house) log(pest) ~ tm + (1|house)			<0.0001 ***
Time Fixed vs. Time*Occupation	log(pest) ~ tm + (1|house) log(pest) ~ tm*occ + (1|house)			0.0005 ***	log(pest) ~ tm + (1|house) log(pest) ~ tm*occ + (1|house)			0.98
Null vs. Time Fixed w/in Occupation								
FW	log(pest) ~ 1 + (1|house) log(pest) ~ tm + (1|house)			0.57	log(pest) ~ 1 + (1|house) log(pest) ~ tm + (1|house)			<0.0001 ***
NFW	log(pest) ~ 1 + (1|house) log(pest) ~ tm + (1|house)			0.28	log(pest) ~ 1 + (1|house) log(pest) ~ tm + (1|house)			<0.0001 ***

**Table 3:** Statistical summary table for weighted neurodevelopmental exposome analysis. Table 3 includes the results of the proportions tests and the mixed effects tests for the unweighted (left, for comparison) and weighted (right) neurodevelopmental exposome analysis. The unweighted analysis is the same as presented above in Table 2. The weighted analysis provides the proportion of FW and NFW households that had potentially neurotoxic pesticide levels greater than the 2005 75th percentile, the difference between FWs and NFWs, and the associated p-value. Additionally, the mixed effects analysis shows which models were compared in the weighted analysis and whether those models were significantly different from one another. ** *p* < 0.01; *** *p* < 0.001.

	Potentially Neurotoxic Pesticides (Unweighted)				Potentially Neurotoxic Pesticides (Weighted)			
Proportions Tests	FW	NFW	Difference (95% CI)	*p*	FW	NFW	Difference (95% CI)	*p*
Proportion Red 2005	0.38	0.11	0.27 (0.08–0.44)	0.01 **	0.43	0.06	0.37 (0.20–0.54)	<0.001 ***
Proportion Red 2011	0.15	0.20	−0.5 (−0.22–0.12)	0.57	0.07	0.04	0.03 (−0.08–0.14)	0.62
	**Potentially Neurotoxic Pesticides (Unweighted)**				**Potentially Neurotoxic Pesticides (Weighted)**			
**Mixed Effects Tests**	**Models**			***p***	**Models**			***p***
Null vs. Time Fixed	log(pest) ~ 1 + (1|house) log(pest) ~ tm + (1|house)			0.75	log(pest) ~ 1 + (1|house) log(pest) ~ tm + (1|house)			0.004 **
Time Fixed vs. Time*Occupation	log(pest) ~ tm + (1|house) log(pest) ~ tm*occ + (1|house)			0.0005 ***	log(pest) ~ tm + (1|house) log(pest) ~ tm*occ + (1|house)			<0.001 **
Null vs. Time Fixed w/in Occupation								
FW	log(pest) ~ 1 + (1|house) log(pest) ~ tm + (1|house)			0.57	log(pest) ~ 1 + (1|house) log(pest) ~ tm + (1|house)			0.001 **
NFW	log(pest) ~ 1 + (1|house) log(pest) ~ tm + (1|house)			0.28	log(pest) ~ 1 + (1|house) log(pest) ~ tm + (1|house)			0.38
